# Learning-Dependent Plasticity of the Barrel Cortex Is Impaired by Restricting GABA-Ergic Transmission

**DOI:** 10.1371/journal.pone.0144415

**Published:** 2015-12-07

**Authors:** Anna Posluszny, Monika Liguz-Lecznar, Danuta Turzynska, Renata Zakrzewska, Maksymilian Bielecki, Malgorzata Kossut

**Affiliations:** 1 Department of Molecular and Cellular Neurobiology, Nencki Institute of Experimental Biology, Warsaw, Poland; 2 Department of Psychology, SWPS University of Social Sciences and Humanities, Warsaw, Poland; 3 Department of Neurochemistry, Institute of Psychiatry and Neurology, Warsaw, Poland; Emory University, UNITED STATES

## Abstract

Experience-induced plastic changes in the cerebral cortex are accompanied by alterations in excitatory and inhibitory transmission. Increased excitatory drive, necessary for plasticity, precedes the occurrence of plastic change, while decreased inhibitory signaling often facilitates plasticity. However, an increase of inhibitory interactions was noted in some instances of experience-dependent changes. We previously reported an increase in the number of inhibitory markers in the barrel cortex of mice after fear conditioning engaging vibrissae, observed concurrently with enlargement of the cortical representational area of the row of vibrissae receiving conditioned stimulus (CS). We also observed that an increase of GABA level accompanied the conditioning. Here, to find whether unaltered GABAergic signaling is necessary for learning-dependent rewiring in the murine barrel cortex, we locally decreased GABA production in the barrel cortex or reduced transmission through GABAA receptors (GABAARs) at the time of the conditioning. Injections of 3-mercaptopropionic acid (3-MPA), an inhibitor of glutamic acid decarboxylase (GAD), into the barrel cortex prevented learning-induced enlargement of the conditioned vibrissae representation. A similar effect was observed after injection of gabazine, an antagonist of GABAARs. At the behavioral level, consistent conditioned response (cessation of head movements in response to CS) was impaired. These results show that appropriate functioning of the GABAergic system is required for both manifestation of functional cortical representation plasticity and for the development of a conditioned response.

## Introduction

Associative learning results in functional changes in the neuronal networks of the brain cortex. In the primary sensory cortex, a phenomenon of enlargement of the representational area of sensory peripheries that receive conditioned stimulus was reported in several laboratories [1–6]. We used classical conditioning involving the whisker-to-barrel field sensory system to induce plasticity of cortical representation of a row of vibrissae and investigate its mechanism [2]. The pairing of vibrissae stimulation with a tail shock results in enlargement of the functional cortical representation of the stimulated whiskers, visualized by [^14^C]2-deoxyglucose mapping.

We previously reported that enlargement of the cortical representation of vibrissae involved in conditioning was NMDA receptor dependent [7] and accompanied by increased excitability of projection neurons [8]. Concurrently, changes in the metabolic pathway for GABA production were observed within the plastic vibrissae representation. The level of GAD67 mRNA and protein was increased, while immunostaining showed a higher density of GAD67-positive neurons and boutons [9,10]. Consistently with the above results, we also found an increased density of GABA immunoreactive cells in layer IV of the cortical representation of conditioned vibrissae [11], and an increase of GABA level in the barrel cortex [12]. Moreover, learning resulted in a 70% increase in the density of inhibitory synapses on dendritic spines, with higher GABA content in their presynaptic terminals [13]. In order to assess whether these changes in GABAergic markers were reflected in altered inhibitory influence imposed on excitatory neurons, we performed intracellular recordings from excitatory neurons involved in conditioned information processing. We found that conditioning resulted in an increase in the frequency of spontaneous inhibitory postsynaptic currents and inhibitory tonic currents in excitatory neurons, with a simultaneous decrease of the latter in fast-spiking inhibitory interneurons [14,15].

All these changes were observed after the end of conditioning and may have been a protective reaction of the cortex to increased excitatory drive caused by incoming CS and UCS signals. On the other hand, a rapid rise of local inhibitory interactions and GABA-ergic input from nucleus basalis may be important for reorganization of cortical circuits by, for example, disinhibition of selected circuits within the cortical column [16].

Although the importance of inhibition for induction of plasticity in sensory cortex was extensively investigated [17], the majority of data concerning adult plasticity was acquired for models involving sensory stimulation or elimination of sensory input. Much less attention was focused on plasticity involved in learning.

The main goal of the current study was to find whether the proper level of GABAergic transmission during sensory training is indispensable for conditioning-induced functional plasticity in the sensory cortex. We found that reducing GABA synthesis or GABAergic signaling in the barrel cortex by blocking GAD activity or GABAARs (GABAA receptors) prevented the development of cortical representation plastic change.

## Materials & Methods

Experiments were performed on 36 six-week-old mice of the C57BL/6J strain. The animals were housed under standard living conditions: in a temperature-controlled room with a light/dark cycle (12h/12h), with food and water available *ad libitum*. All procedures conformed to the European Community Council Directive (86/609/EEC) and were approved by the Local Ethical Commission No.1 in Warsaw. All surgery was performed under Isoflurane anesthesia, and all efforts were made to minimize suffering.

Three experimental groups were used: a group with intracortical injections of 3-MPA, a group with intracortical injections of gabazine, and a control group.

### Classical conditioning

**Fig 1:**
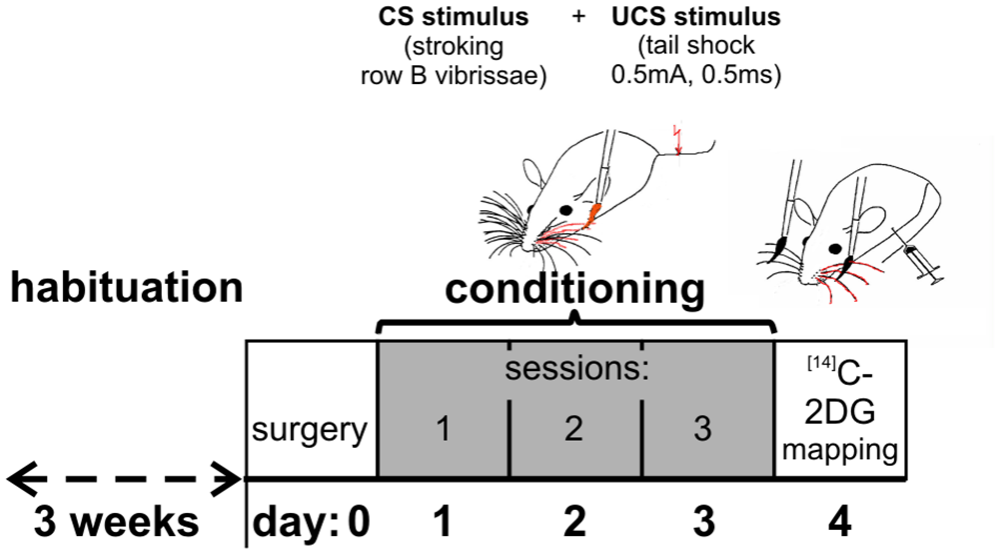
Diagram of the experimental procedure.

Mice were habituated to a restraining holder over a three week period prior to conditioning. The restraining holder was used during experiments to keep the mouse in one place, but allowed for free movements of the head. During conditioning a tactile stimulation of the vibrissae row B on one side of the snout (conditioned stimulus, CS) was paired with a mild tail shock (0.5 mA, 0.5 ms; unconditioned stimulus, UCS). Each pairing consisted of three strokes of vibrissae and tail shock applied at the end of the third vibrissae stroke when the brush was still touching the vibrissae. Mice received 40 stimuli pairings during each conditioning session, and conditioning was accomplished within three sessions (one 10-min session per day) on subsequent days. All conditioning sessions were recorded using a video camera. A schematic representation of the experiment time course is presented in Fig 1.

### Surgery and intracortical injections

Preparatory surgery that facilitated intracortical injections on subsequent days of training was performed 24 hours prior to the first conditioning session. Mice were initially anesthetized with the analgesic Butomidor (Richter Pharma AG, 0.025 ml/mouse) and then inhaled 4% isoflurane (Aerrane, Baxter) throughout the surgery. Localization of the injection site was assigned according to the following coordinates: 2.3 mm posterior to the bregma and 3.4 mm to the right of the sagittal suture. The skull was thinned with a dental drill and a small fragment of the skull was lifted with use of a hypodermic needle and removed to produce a small entrance for the injection capillary. The skin was then sutured, with the last stitch being placed loosely to make for easy access to the injection site on the next day. Immediately after surgery mice received another dose of Butomidor (0.025 ml/mouse).

The mice received intracortical injections under isoflurane anesthesia on each day of conditioning. Compounds were injected through a Hamilton syringe, with a removable glass capillary (tip diameter of 40–50 μm) serving as a needle. The capillary tip was positioned at a depth of 100 μm from the cortical surface. Injections were performed with a microinfusion syringe pump (World Precision Instruments, Inc.) at a speed of 15 nl/min. The injection procedure lasted 10–12 min, and the conditioning session began 20 or 45 min after injection depending on the experimental group. One experimental group of mice received 50 nl-volume injections of 3-MPA at 100 μM-concentration (3-MPA group, n = 5) 20 min before the beginning of the conditioning session. Another group of mice received injections of 50 nl of gabazine, at a concentration of 100 μM (gabazine group, n = 5). Gabazine was administered 45 min before beginning the conditioning session. Mice from the control group received injections of NaCl 0.9% (n = 5). Two animals from the control group received vehicle injections 20 min prior to the conditioning session and three animals received injections 45 min prior.

### [^14^C]2-deoxyglucose (2DG) autoradiography

2DG mapping was performed 24 hours after the last conditioning session. Mice were placed in the restraining holder and all vibrissae except row B on both sides of the snout were trimmed. Mice received injections of [^14^C]2-deoxyglucose (American Radiolabeled Chemicals, ARC, specific activity 55 mCi/mmol; 50 μCi/ 100 g body weight, i.m.) and the remaining row B vibrissae were stimulated with a mechanical stimulator at a frequency of 2 Hz for 30 min. Afterwards, mice received a lethal dose (0.2 ml/mouse) of barbiturate (Vetbutal, Biowet, Pulawy, Poland) and were briefly perfused transcardially with phosphate-buffered saline (PBS, pH 7.4) and 4% paraformaldehyde. The brains were removed and the cortex from two hemispheres was isolated, flattened between two glass slides [18], and fast frozen in heptane chilled to -60°C. The flattened cortex was cut at the cryostat into sections (30 μm) tangential to the cortex surface. Autoradiograms of 2DG labeling on serial brain sections and [^14^C] standards were obtained on Kodak mammography X-ray film after two weeks exposure. After obtaining 2DG autoradiograms, brain sections were counterstained with cresyl violet in order to identify barrels in layer IV of the somatosensory cortex.

### Analysis of autoradiograms

Images of serial brain sections were collected and analyzed with use of Micro Computer Imaging Device (MCID; Imaging Research Inc.), a system for image analysis. Images were corrected for the film background and optical distortions by subtracting the image of a clear region of film. A calibration curve was created on the basis of the absolute gray levels of the [^14^C] standards. The signals on all autoradiograms were within the linear range of this curve. Measurements of the width of the vibrissae row B cortical representations were collected from three sections containing layer IV of the barrel field.

Labeling was quantified as described by Siucinska and Kossut [2]; the criterion for estimating the extent of labeling was that the intensity of 2DG uptake was more than two standard deviations above the mean value of labeling in the surrounding tissue. Statistical analysis was performed using SPSS software (ver. 22, IBM Corp, 2013). The width of the vibrissae row B representations were averaged across three layer IV sections for each animal and hemisphere, and submitted to two-way 2 (Hemisphere) x 3 (Group) mixed design ANOVA. Simple effects analysis with Bonferroni corrections was used as a follow-up analysis.

### Assessment of conditioned response development

Video files recorded during the conditioning sessions were analyzed. As we reported before, the conditioned reaction in this experimental situation is a cessation of head movements in response to the conditioned stimulus [19]. Mice naturally turn the head towards the stimulator touching the vibrissae. After a few trials when stroking the vibrissae is paired with tail shock, this reaction ceases; this is akin to freezing observed in other fear conditioning paradigms. The total number of head movements in response to the conditioned stimulus, (CS-head movements) was computed for each animal and session. Only head movements performed in response to the conditioned stimulus were included in the analysis and the results are presented as a number of trials in which a head turn was present in reaction to the CS. Statistical comparisons within particular experimental groups were performed using One Way ANOVA with Tukey's Multiple Comparison Test, while for comparisons between groups we used Two Way ANOVA with Bonferroni Post Test. All statistical analysis were done using GraphPad Prism 5 software (GraphPad Software, Inc.)

### High-performance liquid chromatography (HPLC)

In the preliminary experiments, injections of 100 μM 3-MPA (50 nl) into the right hemisphere were done according to the previously defined coordinates (n = 10). NaCl 0.9% (50 nl) was injected into the contralateral (left) hemisphere at the corresponding localization. Samples of barrel cortex from the right and left hemispheres were collected 20 min after 3-MPA injection. Additionally, samples of the cortex from the left hemisphere anterior to the barrel cortex (non-injected cortex) were collected from five mice. The barrel cortex was dissected on ice from a fresh flattened cortical tissue using a 2-mm-diameter template according to the method described by Strominger and Woolsey [18]. Samples of tissue from the barrel cortex were collected and immediately frozen on dry ice. The level of gamma-aminobutyric acid (GABA) as well as other amino acids (glutamine, glutamic acid, glycine, aspartic acid, alanine), and the organic acid taurine was assessed. HPLC analysis of amino acids and taurine was performed as previously described [12]. Statistical analysis was performed using GraphPad Prism 5 software (GraphPad Software, Inc.) with paired two-tailed t-test.

### Gabazine dose establishment

Two gabazine doses were tested, both of the same volume (50 nl) but at two concentrations: 1000 μM (n = 3) or 100 μM, (n = 8). Gabazine was injected intracortically at the stereotaxic coordinates defined above (see subsection *Surgery and intracortical injections*). 2DG mapping of the vibrissae row B was performed 45 min after gabazine injection. The procedure of 2DG mapping was the same as described in the subsection *[* ^*14*^ *C]2-deoxyglucose autoradiography*. For the lower dose of gabazine, we performed quantitative analysis of the stimulated row representation labeling. Comparison of the width of the representation within control and injected with gabazine hemisphere was performed using GraphPad Prism 5 software (GraphPad Software, Inc.) with paired two-tailed t-test.

## Results

### Plasticity of the vibrissae cortical representation

**Fig 2:**
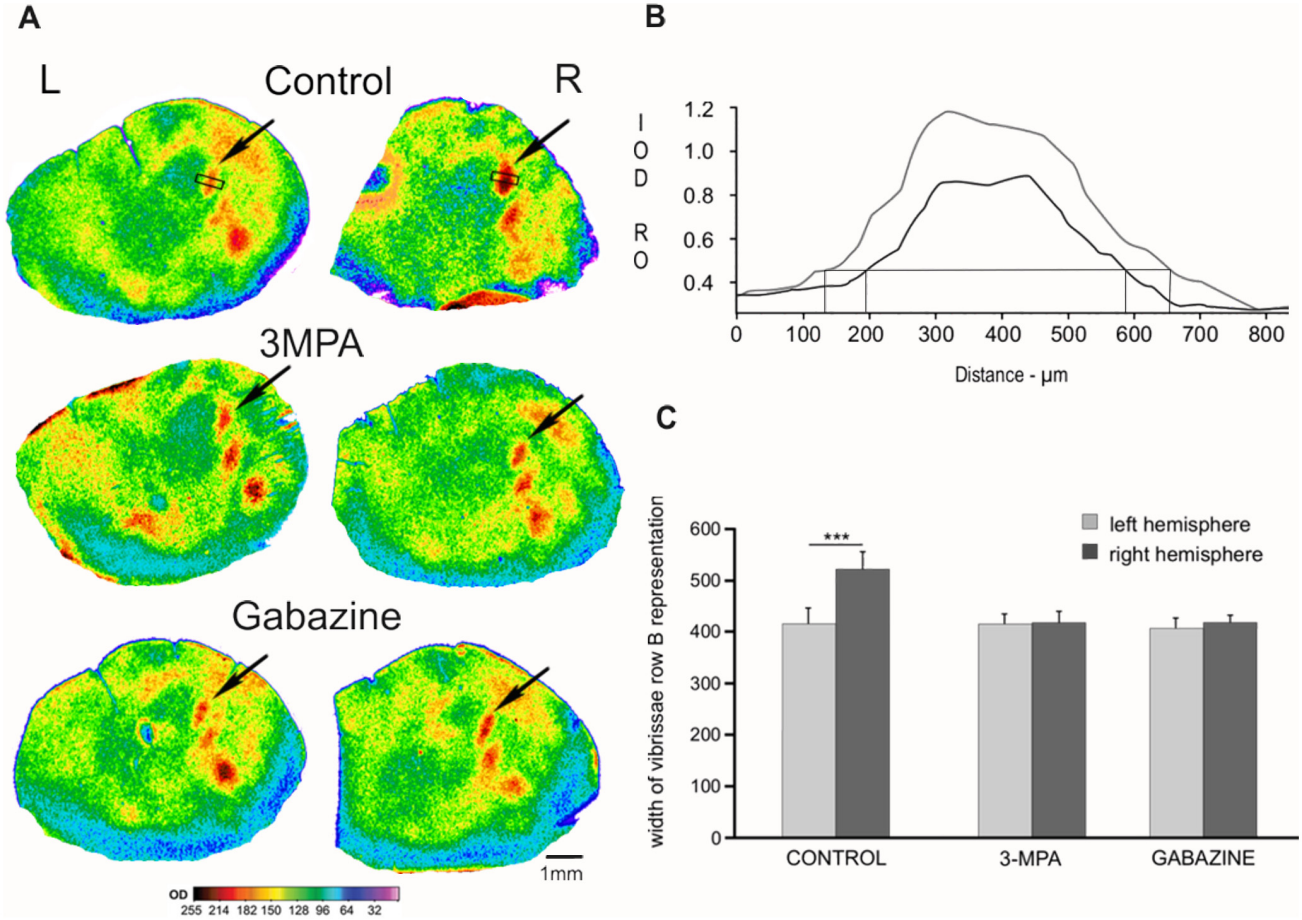
Effect of 3-MPA or gabazine on the conditioning-induced widening of the row B cortical representation. (A) Pairs of 2DG autoradiograms of the left and right hemisphere tangential brain sections from barrel cortex layer IV, from individual mice of control group, 3-MPA group, and gabazine group. N = 5 in each group. In each group the left hemisphere (L) served as a control and right hemisphere (R) was involved in the conditioning and injected with compounds: 0.9% NaCl (in the control group), gabazine, or 3-MPA (in experimental groups). Arrows point to the representation of stimulated row B. (B) Plot of the 2DG-labeling intensity (presented in arbitrary units of optical density) profile of the row B representation from the autoradiograms of right and left hemispheres from the control mice. (C) Plot of the width of vibrissae row B cortical representation in both brain hemispheres, means ± SD (left hemisphere—light gray, right hemisphere—dark gray; Bonferroni Post Test: *** p< 0.0001).

Stimulation of a row of vibrissae during 2DG incorporation results in a band of higher 2DG uptake, extending throughout the cortical depth and centered on the cognate row of barrels [20]. This is a functional cortical representation of the stimulated row of vibrissae. Similarly to our earlier observations, we also observed that as a result of conditioning, the 2DG labeled functional representation of the”trained” row of vibrissae expanded in the control, saline injected group (Fig 2). ANOVA results revealed that main effects of Group (F_(2, 12)_ = 15.96, p < 0.001, η_p_^2^ = 0.73) and Hemisphere (F_(1, 12)_ = 74.44, p < 0.00001, η_p_^2^ = 0.86) were qualified by a robust interaction (F_(2, 12)_ = , p < 0.00001, η_p_^2^ = .90). Simple effects of hemisphere were computed for each group as a follow up analysis. As expected, widening of the vibrissae row B representation in the hemisphere involved in conditioning was observed in the control group only (F_(1, 12)_ = 185.87, p < 0.000001, η_p_^2^ = 0.94). The mean width of the vibrissae row B representation involved in conditioning was 513 ± 32 μm, which was 25% greater than the contralateral, control hemisphere width of 411 ± 22 μm.

Application of GAD activity blocker 3-MPA prevented the development of cortical plastic change, as indicated by non-significance of the simple effect of hemisphere (F_(1, 12)_ = 1.84, p = 0.201, η_p_^2^ = 0.13). In the 3-MPA group, the mean width of the row B vibrissae cortical representation in the hemisphere involved in conditioning and injected with 3-MPA was 410 ± 11 μm, and in the contralateral, control hemisphere it amounted to 400 ± 15 μm (Fig 2).

Gabazine injections performed before each conditioning session also counteracted conditioning-induced widening of the vibrissae row B cortical representation. The mean width of labeling in the hemisphere involved in conditioning and injected with gabazine was 405 ± 21 μm, whereas in the contralateral, control hemisphere it was 406 ± 8 μm (Fig 2). Simple effect of hemisphere was again non-significant (F_(1, 12)_ = 0.002, p = 0.97, η_p_^2^ < 0.001).

Coherent results were obtained in the simple effects analysis of Group. No reliable differences were observed in the control hemisphere (F_(2, 12)_ = 0.57, p = 0.578, η_p_^2^ = 0.09). The same analysis performed for the hemisphere involved in conditioning revealed significant effects (F_(2, 12)_ = 34.00, p < 0.0001, η_p_^2^ = 0.85). Row B cortical representations were significantly wider in the control group when compared with two other conditions, as confirmed by Bonferroni post-hoc tests (p < 0.0001).

### Development of conditioned response

**Fig 3:**
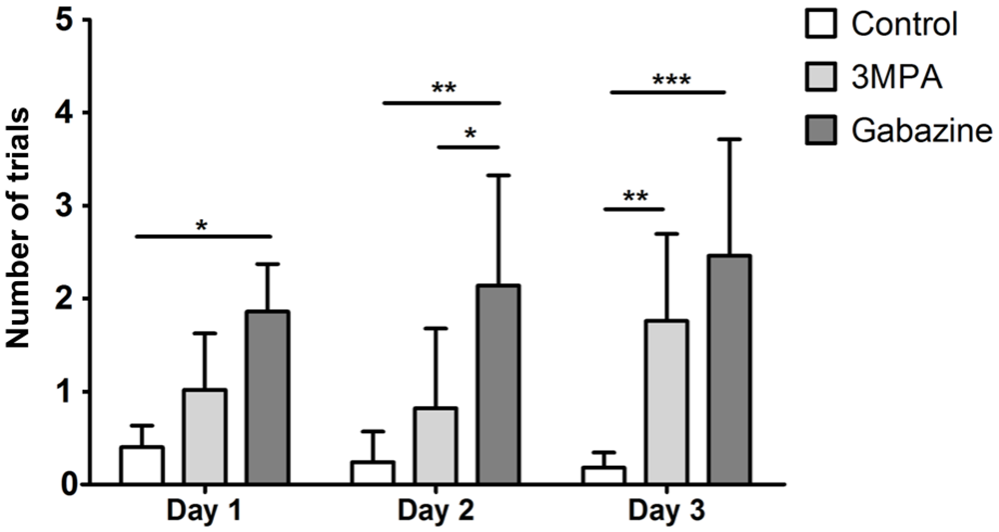
Comparison of the behavioral response in subsequent days of conditioning for all experimental groups. Number of trials in which a head turn was present in reaction to the CS, means ± SD (Bonferroni Post Test: * p< 0.01, ** p< 0.001, *** p< 0.0001).

Effectiveness of the conditioning was defined as a monotonic decrease in the number of head movements in response to CS across all three days of training. This is a response akin to freezing, observed after application of the UCS alone and CS alone 24 hrs after conditioning [19]. Two Way ANOVA analysis of conditioned response in all three groups (control, with 3-MPA and gabazine injection) during three consecutive sessions (day1-day3) revealed significant effect of treatment (F_(2,36)_ = 22.71; P<0.0001). Conditioned response was significantly better expressed in the control than in the gabazine group and this effect was visible already from the first training session (Day 1: p< 0.01; Day 2: p< 0.001; Day 3: p<0.0001; Bonferroni Post Test). The difference between controls and the 3-MPA group was less evident and was present only in the third training session (Day 3: p< 0.0001; Bonferroni Post Test) (Fig 3).

**Fig 4:**
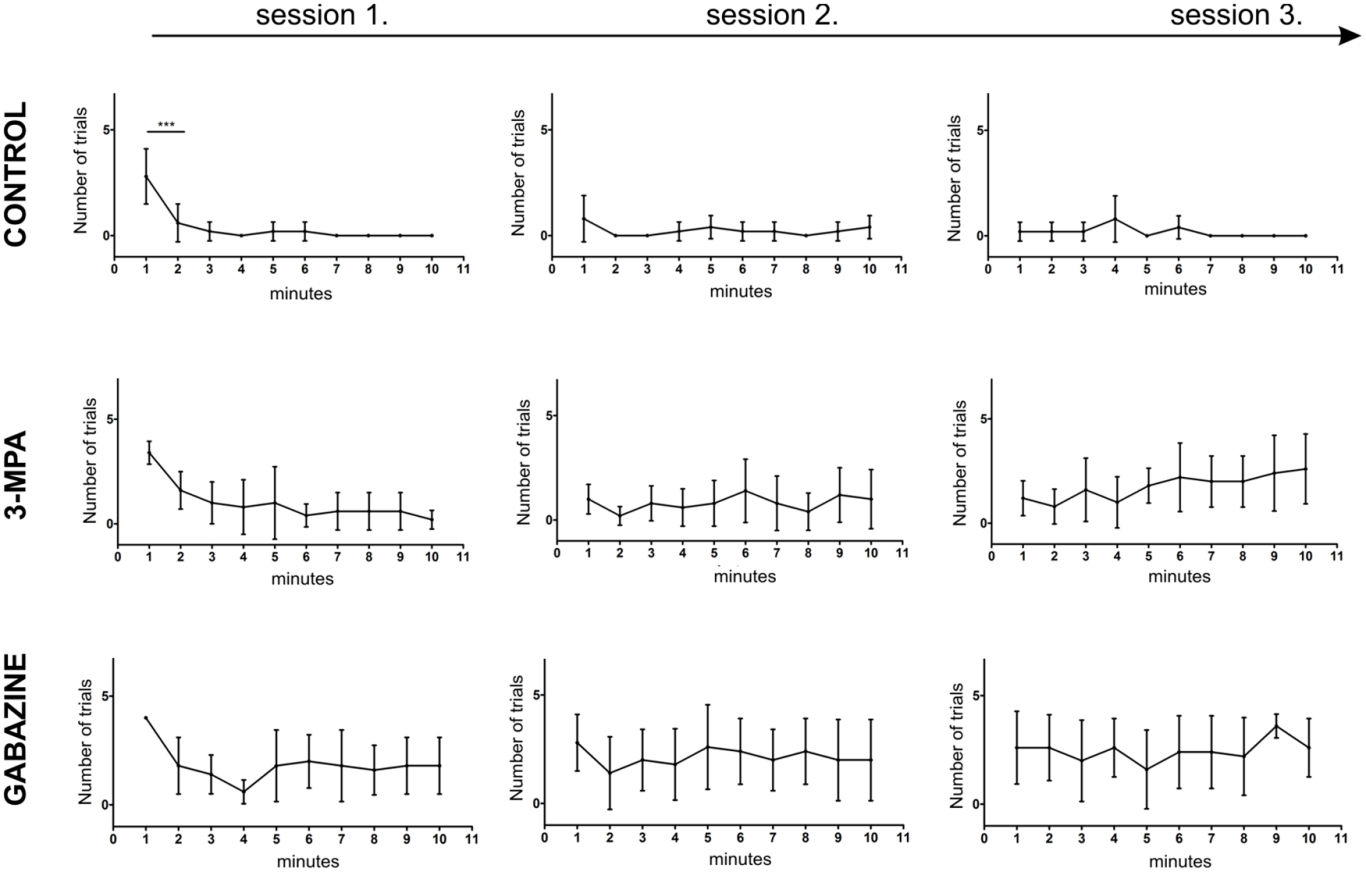
Effect of gabazine or 3-MPA on the learning-induced changes in the number of head movements in response to vibrissae stroking (conditioned stimulus) during the course of conditioning. Means (± SD) of the number of head movements at subsequent minutes of the conditioning sessions in the control group, 3-MPA group, and gabazine group (Tukey's Multiple Comparison Test: *** p< 0.001).

**Fig 5:**
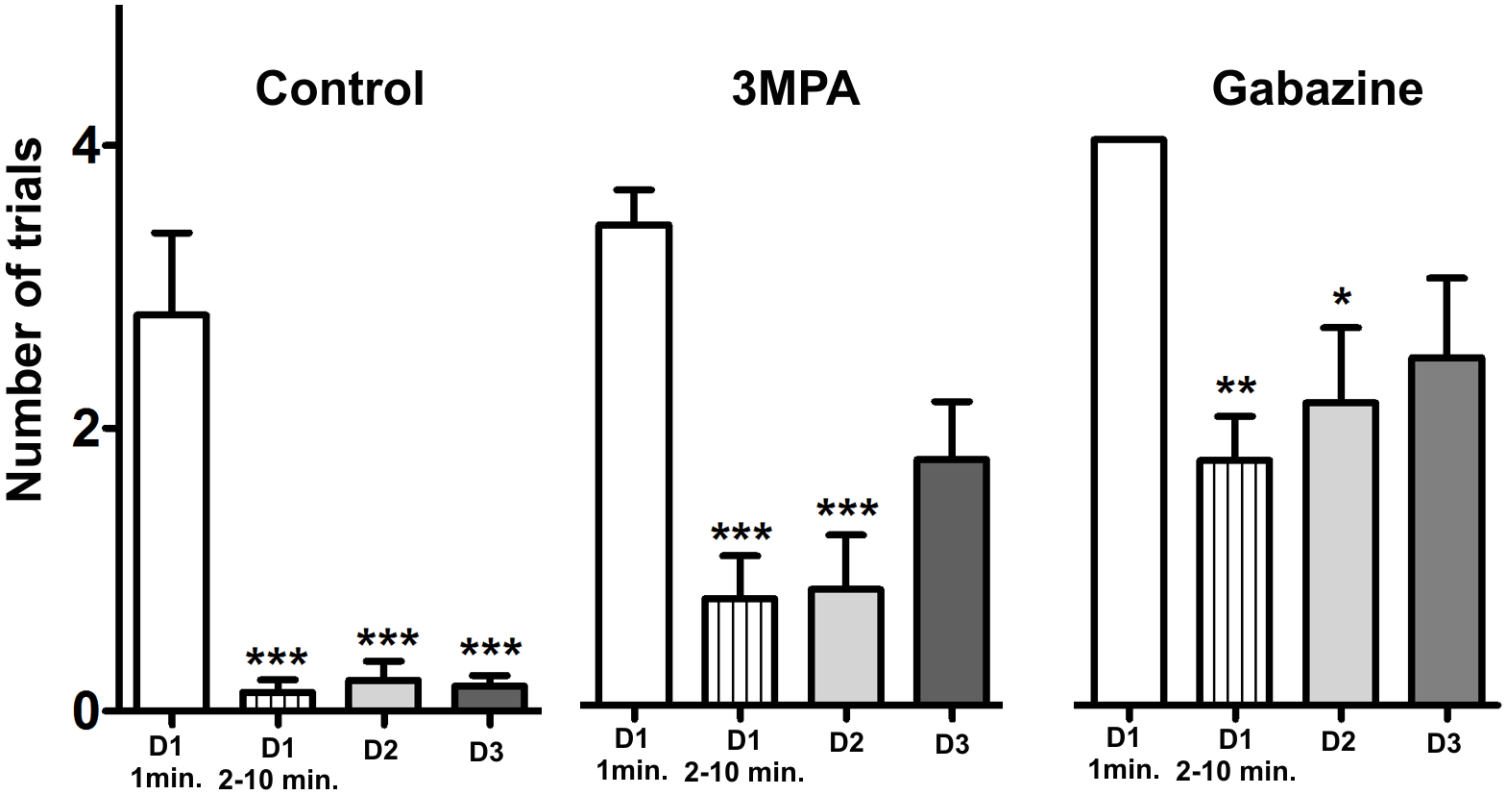
Effectivness of learning throughout the conditioning. Within group comparison of the conditioned responses between the first minute of the 1st day of conditioning (D1 1 min.) and the rest of the first session (D1, 2–10 min.), the whole second (D2) and whole third sessions (D3), means ± SD (Tukey's Multiple Comparison Test: * p< 0.01, ** p< 0.001, *** p< 0.0001).

Detailed analysis of learning curves within groups using One Way Anova with Tukey's Multiple Comparison Test revealed that in control animals, the first significantly lower response to conditioned stimulus was present as early as in the second minute of the training (F_(4,49)_ = 13.8; P< 0.0001). This effect was absent in both 3-MPA and gabazine injected animals (Fig 4). Therefore, to verify the effectiveness or ineffectiveness of conditioning during consecutive days of the training, we compared the behavioural response present during the first minute of the training (D1, 1 min.) to the rest of the first session (D1, 2–10 min.) as well as to the whole second (D2) and whole third sessions (D3). In the control group, the effectiveness of conditioning was confirmed from the first training session and was stable across the whole training period (F_(11,59_ = 13.41; P< 0.0001; One Way Anova with Tukey's Multiple Comparison Test) (Fig 5; left panel). 3-MPA injected mice showed the effects of conditioning during the first and second training sessions (P< 0.0001; One Way Anova with Tukey's Multiple Comparison Test) however, on the third day of training the response to CS was erratic (Fig 5; middle panel). Similar effect, although with different dynamics, could be observed in gabazine injected animals (Fig 5; right panel). The presented data mean that effective behavioural changes throughout the whole period of the training are necessary for the manifestation of the conditioning-induced plastic change.

### Control experiments: estimation of 3-MPA injection effectiveness and dose effect of gabazine

During preliminary experiments we estimated the location and extent of the injections using 50 nl of Green Fast dye and we found that 20 min after injection the dye stained most of the posteromedial barrel sub-field covering completely row B of barrels. Moreover, the staining was confined to the cortex never reaching the subcortical tissue.

**Fig 6:**
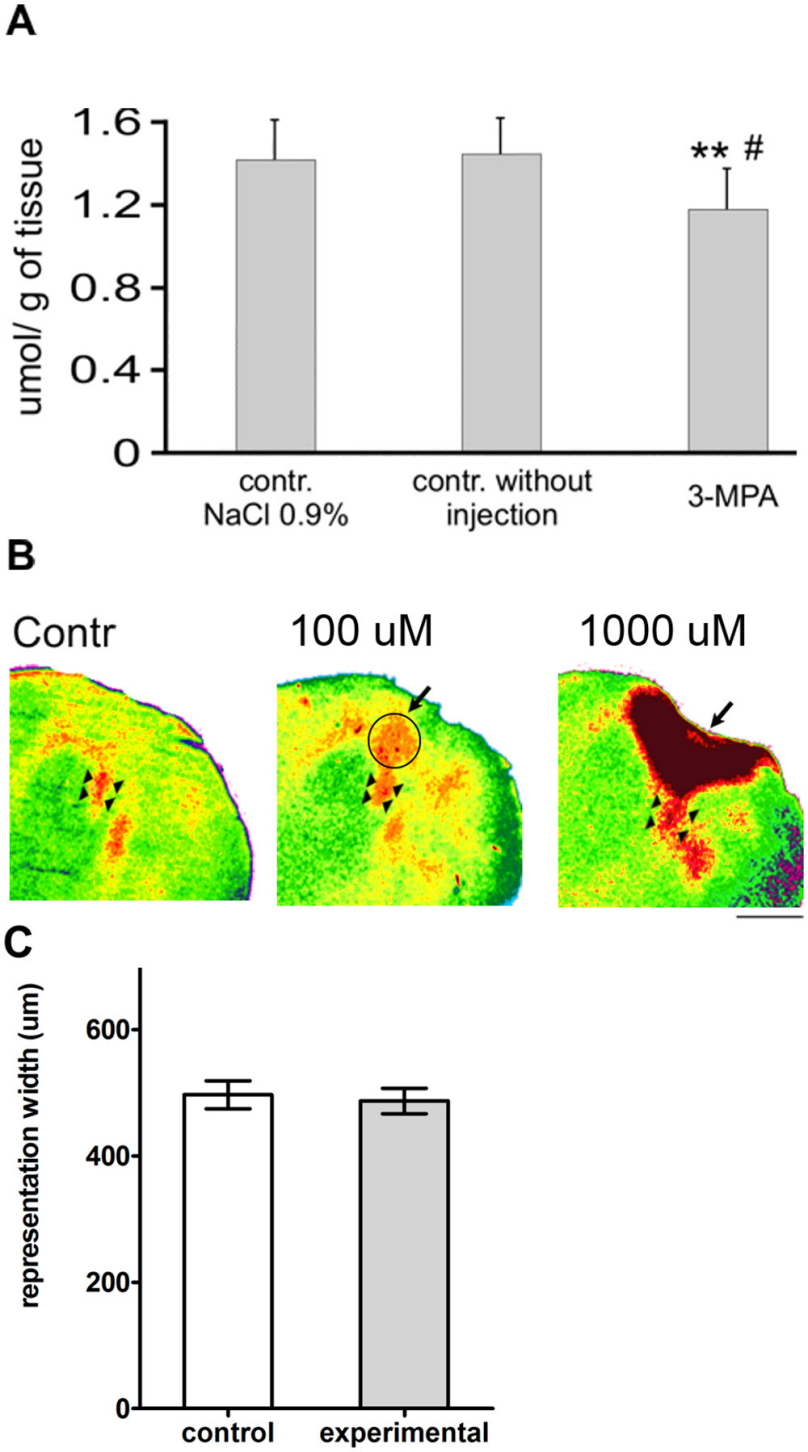
Results of preliminary studies on the effect range of used compounds. (A) Effect of 3-MPA on level of GABA in the barrel cortex 20 min post injection (the time designed for the beginning of conditioning session) assessed with HPLC. Comparisons with use of paired two-tail t-test were performed between samples of the barrel cortex injected with 3-MPA and samples of the control barrel cortex injected with 0.9% NaCl (**p< 0.004) or the not injected control cortex (# p< 0.02, n = 5). (B) Pseudocoloured 2DG autoradiograms of tangential sections of the cortex, posteromedial quadrant of the section through layer IV. Effects of two doses of gabazine on functional mapping of vibrissae cortical representation with 2DG, performed 45 min after gabazine injection. Arrowheads point to the labeling evoked by stimulation of row B whiskers, much wider after higher, but not after lower dose of gabazine. Arrows point to the injection site behind the barrel field. In the middle panel (lower dose of gabazine) circle indicates the activation around the injection site. Higher dose of gabazine (right panel) resulted in extensive activation around the injection site. (C) Quantification of the effect of lower dose of gabazine on the width of the row B whisker cortical representation (n = 8). Comparison with paired two-tail t-test (no significance).

The effect of a chosen 3-MPA dose on the level of GABA in the barrel cortex was estimated by HPLC. Assessment was performed at the time designed for the beginning of the conditioning session (20 min post injection). GABA level in samples of barrel cortex with 3-MPA injection (mean value 1.179 ± 0.196 μmol/g of tissue) was 15% lower than in the control barrel cortex with an injection of 0.9% NaCl (mean value 1.395 ± 0.202 μmol/g of tissue; paired two-tailed t-test p< 0.004) and 18% lower than in the non-injected cortex (mean value 1.442 ± 0.172 μmol/g of tissue; paired two-tailed t-test p< 0.02; Fig 6A). In comparisons between samples of barrel cortex injected with 3-MPA, 0.9% NaCl, or samples of non-injected cortex there were no differences in the level of amino acids other than GABA (glutamine, glutamic acid, glycine, aspartic acid, alanine) and organic acids (taurine).

The effect of two different gabazine doses (50 nl at concentrations of 1000 μM or 100 μM) was estimated with 2DG functional mapping of vibrissae row B representation at the time designed for the beginning of the conditioning sessions in the gabazine group, i.e. 45 min after injection, in control-non-trained animals. After reduction of inhibition with a gabazine dose of 50 nl at a concentration of 1000 μM, the cortex around injection site was heavily labeled, indicating high activation (Fig 6B). Stimulation of row B vibrissae evoked robust 2DG labeling that extended far beyond vibrissae row B representation. After application of the same volume of gabazine, but at a concentration of 100 μM, increased activation was observed in cortex around the injection site but there was no increment in the extent of row B labeling in the barrel cortex in comparison to the control hemisphere (487.2 ± 20.17μm vs. 496.9 ± 22.19μm; p = 0.6; T-test) (Fig 6B and 6C). Therefore this dose was chosen for further experiments.

## Discussion

We found that transient reduction of GABA level and GABAergic transmission locally in the area of the barrel cortex, blocked plastic changes induced by conditioning. Intracortical injections of low doses of gabazine or 3-MPA into the barrel cortex before each conditioning session prevented learning-induced widening of the cortical representation of row B vibrissae involved in conditioning, whereas this widening was observed in control mice that received vehicle injection. At the behavioral level, consistent performance of conditioned response in mice receiving gabazine or 3-MPA was impaired.

Activity-induced enhancement of inhibitory synaptic transmission has been shown in several brain regions of the mammalian brain [21–23]. In our previous reports we showed a conditioning-induced increase in many markers related to the GABAergic pathway, as well as functional upregulation of the inhibitory system [9–15]. Since these changes were assessed 24 hours after conditioning, we could not classify them either as necessary for plastic changes or as the result of a newly established balance between excitatory and inhibitory systems, a consequence of the plastic change. In the present study, the idea was to interfere with GABAergic signaling during conditioning in order to assess whether appropriate level of GABAergic transmission is indispensable for conditioning-induced functional plasticity in the barrel cortex. Different forms of plasticity at GABAergic synapses occurring as a consequence of excitatory activity were based on either presynaptic or postsynaptic mechanisms [24,25]. Therefore, we decided to interfere presynaptically with GABA synthesis and postsynaptically with GABA receptors activity to assess the involvement of both mechanisms in conditioning induced plasticity. For transient reduction of GABA production we chose 3-MPA, an inhibitor of GAD activity acting fast and with a moderate time of reversal [26,27]. Its efficacy was confirmed by Engel et al. [27], who showed a reduction of inhibitory postsynaptic currents in CA3 pyramidal cells in slice cultures as the effect of 3-MPA administration. 3-MPA action begins as soon as 3 min and is reversed after 30–60 min [28–30]. In the preliminary studies we established that the level of GABA in tissue samples of barrel cortex was significantly but moderately (by 15%) reduced 20 minutes after intracortical 3-MPA injection. The same concentration of 3-MPA was used in the experiments by Harauzov et al. [31]. They showed that even continuous infusion of the drug for one week in the visual cortex resulted only in modest increase in spontaneous activity with no decrease in cell responsiveness. Therefore, we do not expect that one injection of 3-MPA on every training day could strongly alter neuronal network excitability.

In order to investigate the impact of postsynaptic mechanisms we used gabazine, an antagonist of GABAARs. Although a question can arise if lack of plasticity could result from massive activation of the circuitry in the first minutes of gabazine infusion, it seems unlikely for several reasons. First of all the chosen dose of gabazine was a modest one in comparison to the literature data [32–35]. Thiele et al. [36] applied much higher dose of gabazine (3 mM) into a monkey cortex and it did not substantially affected firing rate of cortical neurons. In our experiments the injection site was located outside the barrel field, therefore concentration resulting from diffusion of the drug to whisker representation area was certainly much lower than in the injection site. It neither evoked significant change in the 2DG uptake nor produced noticeable abnormalities in the animal’s behavior before the training.

Our results demonstrate that cortical plasticity evoked by classical conditioning engages GABAergic transmission mechanisms, since both strategies we used to alter inhibitory signaling resulted in plasticity impairment. We previously observed that in aged animals after a three-day conditioning GABA level is not elevated and plastic change is absent [12,37]. Both studies, the present and by Liguz-Lecznar et al. [12], show that exogenously or naturally induced disruption of the balance between GABAergic and excitatory transmission results in cortical plasticity impairment.

We found that enlargement of the representational area for the conditioned stimulus requires unimpaired inhibitory transmission during the time of associated stimuli presentation. It seems paradoxical, as decreased inhibition facilitates LTP induction [38] increases receptive fields [39,40] and accompanies experience-dependent plasticity [41]. Besides, multiplying of cells responding to a stimulus mostly connotes with excitation in the neuronal networks. However, in several learning paradigms increased GABAergic system activity accompanied increased excitability in principal neurons in the sensory cortex [14,42–44]. In response to stimulation, inhibitory system tends to upregulate [45]; it was demonstrated that prolonged whisker stimulation increases the number of GAD immunoreactive puncta and the number of inhibitory synapses in the barrel cortex [46,47]. In the case of conditioning, increased excitation of the principal neurons has to be organized into time-ordered neuronal firing, and this is shaped by interneuronal activity in response to neuromodulatory inputs [48,49].

As the PV+ and SOM+ interneurons constitute the two most numerous groups among GABAergic interneurons of the brain cortex and their axonal endings innervating excitatory projection neurons diverge extensively, they are the most influential inhibitory interneuron populations in the cortical networks. It is therefore plausible that a great part of the effect of GABAergic agents applied in our experiments is due to attenuation of activity of PV+ and SOM+ interneurons. Recently we have confirmed the involvement of SOM+ interneurons in the learning-induced cortical plasticity in mice [50]. According to Chiu et al. [51], the inhibitory synapses of spines in cortical layer IV, which we find in significantly increased numbers after conditioning [13], are from SOM interneurons. The inhibition-regulated processes occurring during the time of associative learning, indicate that complex modulation within the interneuron network as well as in reciprocal transmission between excitatory projecting neurons and inhibitory interneurons provide the shift in the cortical network performance required for acquisition of information [52–54]. This shift may leave lasting traces, the evidence of which we find 24 h after conditioning.

Pharmacological disruption of inhibitory transmission impairs the development of cortical plastic change and interferes with the persistence of the behavioral effect of conditioning, possibly affecting the process of consolidation. In our control and experimental groups the effect of the first few pairings of CS and UCS resulted in the expected cessation of head movements in response to CS (Fig 3). However, in the following sessions of training, the control mice remained immobile while in both experimental groups the response fluctuated, with considerable head mobility. From our previous studies we know that behavioral response and manifestation of cortical plasticity are not progressing simultaneously. It is known that learning in a delay fear conditioning task depends on subcortical circuits [55,56], however, it also affects the cortex [42,48], but these two processes may have disparate dynamics. Therefore, alterations of inhibitory interactions in the cortex may influence them differently. Nonetheless, our results demonstrate that normal functioning of the GABAergic system is required equally for induction of learning-dependent cortical plasticity and for the maintenance of the conditioned response.
